# Association mapping for root architectural traits in durum wheat seedlings as related to agronomic performance

**DOI:** 10.1007/s11032-014-0177-1

**Published:** 2014-11-27

**Authors:** Maria Angela Canè, Marco Maccaferri, Ghasemali Nazemi, Silvio Salvi, Rossella Francia, Chiara Colalongo, Roberto Tuberosa

**Affiliations:** 1 Department of Agricultural Sciences (DipSA), University of Bologna, Viale Fanin 44, 40127 Bologna, Italy; 2 Department of Plant Production, I.A.U. Haji abad Branch, Haji abad, Iran

**Keywords:** Association mapping, *Triticum turgidum* L. subsp. *durum* (Desf.) Husn., QTL, Root architecture, Yield, Drought, Agronomic performance, Root number, Seminal root angle

## Abstract

Association mapping provides useful insights on the genetic architecture of quantitative traits across a large number of unrelated genotypes, which in turn allows an informed choice of the lines to be crossed for a more accurate characterization of major QTLs in a biparental genetic background. In this study, seedlings of 183 durum wheat elite accessions were evaluated in order to identify QTLs for root system architecture (RSA). The QTLs identified were compared with QTLs detected for grain yield and its component traits, plant height and peduncle length measured in a previous study where the same accessions were evaluated in 15 field trials with a broad range of soil moisture availability and productivity (Maccaferri et al. in J Exp Bot 62:409–438, 2011). The following RSA features were investigated in seedlings at the four-leaf stage: seminal root angle, primary root length, total root length, average root length, root number and shoot length. Highly significant differences among accessions were detected for all traits. The highest repeatability (*h* ^2^ = 0.72) was observed for seminal root angle. Out of the 48 QTLs detected for RSA, 15 overlapped with QTLs for agronomic traits and/or grain yield in two or more environments. The congruency of the effects of RSA traits and agronomic traits was evaluated. Seminal root angle and root number appear the most promising traits for further studies on the adaptive role of RSA plasticity on field performance in environments differing for water availability. Our results provide novel insights on the genetic control of RSA and its implications on field performance of durum wheat.

**Electronic supplementary material:**

The online version of this article (doi:10.1007/s11032-014-0177-1) contains supplementary material, which is available to authorized users.

## Introduction

The fast rise in global food demand coupled with the increasing unpredictability of weather conditions consequent to climate change require the release of cultivars with higher yield potential and able to maintain acceptable yield levels and quality under a broad range of environmental conditions. In view of the quantitative nature of the traits governing yield and yield stability, effectively meeting this formidable challenge will require a multidisciplinary approach based upon both conventional and genomics-assisted breeding practices. Accordingly, major efforts are underway to identify loci (genes and QTLs) for morpho-physiological traits that control yield potential and yield stability, particularly in cereal crops grown across regions characterized by a broad range of water availability (Tuberosa et al. 2007; Fleury et al. 2010; Uga et al. 2013). An example is provided by the Mediterranean Basin, where durum wheat (*Triticum durum* Desf.) is grown in a range of conditions varying from favorable environments to dryland areas characterized by frequent drought episodes and high temperature stresses, mainly during grain filling (Loss and Siddique 1994; Royo et al. 2010; Maccaferri et al. 2011). Under such conditions, the evaluation of a suitable set of genotypes provides valuable leads for the identification of drought-adaptive traits (Blum 1988; Grando and Ceccarelli 1995; Passioura 2002; Richards 2006; Araus et al. 2008; Reynolds and Tuberosa 2008; Passioura and Angus 2010; Royo et al. 2010; Tardieu and Tuberosa 2010) and the underlying QTLs (Sanguineti et al. 2007; Mathews et al. 2008; Maccaferri et al. 2011; Bennett et al. 2012; Bai et al. 2013; Graziani et al. 2014).

 In this context, the study of root architectural system (RSA) features/QTLs as related to crop performance can help to identify proxy traits for enhancing adaptation to different soil properties, moisture conditions, nutrient concentration, etc. (Bacon et al. 2003; Yu et al. 2007; Hochholdinger and Tuberosa 2009; Obara et al. 2010; Sharma et al. 2011; Tuberosa 2012; Lynch 2013; Uga et al. 2013). For example, deep roots might provide a higher protection against dehydration by extracting water stored in deep soil horizons (Ehdaie et al. 2003; Manschadi et al. 2006, 2010; Lilley and Kirkegaard 2007; Hammer et al. 2009; Wasson et al. 2012; Uga et al. 2013). Therefore, identifying and introgressing alleles for deeper rooting in shallow-rooted, drought-susceptible cultivars (Grando and Ceccarelli 1995; Steele et al. 2007, 2008; Ehdaie et al. 2010; Uga et al. 2013) is a desirable approach, as underlined by the ‘steep, cheap and deep’ ideotype recently proposed by Lynch (2013).

The evaluation of RSA features directly in the field is very difficult, expensive and time-consuming, especially when dealing with the large number of plants and genotypes required for QTL analysis, particularly with target traits of low heritability (Richards 2008; Christopher et al. 2013). Moreover, field screening is usually destructive and leads to a substantial loss of the geometry of the root (Nagel et al. 2009). In this respect, it has been reported that adult geometry of the root is strongly related to seminal root angle (SRA), with deeply rooted wheat genotypes showing a narrower SRA, while genotypes with a shallower root system show wider SRA (Manschadi et al. 2008).

Different systems have been adopted to enable an early screening of RSA traits in wheat (Kubo et al. 2007; Sanguineti et al. 2007; Nagel et al. 2009; Munns et al. 2010; Ren et al. 2012; Bai et al. 2013; Christopher et al. 2013; Liu et al. 2013; Watt et al. 2013). In these cases, the assumption is that genotypes that differ in RSA at an early stage would also differ in the field at stages when nutrient and/or water capture is most critical for grain yield.

Among the possible approaches for the functional dissection of quantitative traits, association mapping (AM) has been developed as an alternative to traditional bi-parental linkage mapping to identify associations between phenotypic values of target traits and molecular markers (Ersoz et al. 2007; Sorrells and Yu 2009). In this study, the set of elite durum wheat accessions previously tested for yield and other agronomic traits in 15 field trials carried out by Maccaferri et al. (2011) across a broad range of Mediterranean environments was evaluated at an early growth stage in order to map RSA–QTLs and verify their effects on grain yield and other agronomic traits.

## Materials and methods

### Plant material

The panel of 183 elite accessions of durum wheat included cultivars and breeding lines selected in Mediterranean countries (Italy, Morocco, Spain, Syria and Tunisia), Southwestern USA and Mexico that were released from the early 1970s up to the late 1990s. The panel included also ‘founder genotypes’ used as parents in breeding programs throughout the Mediterranean Basin and at International CGIAR Centers (CIMMYT and ICARDA). The accessions were chosen according to their pedigree and highly related accessions were excluded. Accessions showing large differences in heading date were excluded to limit possible bias of phenology in the interpretation of the results pertaining to the agronomic traits. A detailed phenotypic and molecular characterization of the panel was previously reported in Maccaferri et al. (2006, 2010, 2011).

### Root morphology evaluation

Root morphology was evaluated according to the protocol first described by Bengough et al. (2004), then modified by Sanguineti et al. (2007) and further modified in the present work. For each genotype, 15 seeds were weighed, then sterilized in a 1 % sodium hypochlorite solution for 10 min, rinsed thoroughly in distilled water and placed in Petri dishes at 28 °C for 24 h. Then, eight homogeneous seedlings with normal seminal root emission were positioned spaced 5 cm from each other on a filter paper sheet placed on a vertical black rectangular (42.5 × 38.5 cm) polycarbonate plate for root obscuration. Distilled water was used for plantlets’ growth. Each experimental unit included six plantlets, since the two external ones were considered as border plantlets and, as such, discarded. RSA traits were measured in plantlets that were grown for 9 days at 22 °C under a 16-h light photoperiod. The experiment was conducted according to a randomized complete block design, with three replications in time.

The following traits were investigated on a single-plant basis: spread of seminal root angle (SRA), first measured at 3.5 cm from the tip of the seeds as the distance between the two external roots of each plantlet and then converted to degrees, primary root length (PRL), total root length (TRL), total number of roots (TRN), average root length (ARL), and shoot length (SL).

Due to the high number of genotypes under evaluation, the accessions were divided into sets of 25–30 accessions each hereafter reported as blocks. In order to account for possible differences in growth rate among blocks, blocking was taken into account in the subsequent analysis of variance (ANOVA) and a linear adjustment for block effect was carried out. Cultivar Meridiano was also repeated as internal check in every block.

RSA traits were measured on plantlets’ images using the software SmartRoot^®^ (Lobet et al. 2011) for all the traits, except for SRA and SL that were measured manually.

### Field data

Details and results of the agronomic performance of the panel of accessions were reported in Maccaferri et al. (2011). Briefly, the 183 accessions were tested in 15 field trials carried out during two growing seasons (2003/2004 and 2004/2005) in six countries (Italy, Lebanon, Morocco, Spain, Syria and Tunisia) and in some cases at two water regimes (rainfed and irrigated). Each trial has been coded according to the country (first three letters of each code), the water regime (with ‘r’ and ‘i’ standing for rainfed and irrigated trial, respectively) and the year (with 04 and 05 standing for 2004 and 2005, respectively) in which they were conducted. More in detail, three trials were carried out in Italy (Itl1-r04 in Cadriano, 44°33′N and 11°24′E; Itl2-r04 and Itl2-r05 in Cerignola, 41°28′N and 15°84′E), four in Lebanon (Lbn-r04, Lbn-i04, Lbn-r05 and Lbn-i05 in Rayack, 33°51′N and 35°59′E), two in Morocco (Mrc-r04 and Mrc-i04 in SidiElaydi, 31°15′N and 7°30′W), two in Spain (Spn1-r04 in Gimenells, 38°56′N and 0°29′E; Spn2-r05 in Granada, 37°15′N and 3°46′E), two in Syria (Syr-r05 and Syr-i05 in Tel Hadya, 36°56′N and 36°04′E) and two in Tunisia (Tns-r05 and Tns-i05 in Kef, 36°14′N and 8°27′E). Each field trial was characterized for the main environmental conditions, namely temperature, water availability and soil moisture. For the present study, a re-analysis of agronomic traits was performed with a new genetic map assembled at University of Bologna (Maccaferri et al. 2014a). In particular, the analysis focused on grain yield (GY), thousand kernel weight (TKW), number of grains per square meter (KPSM), grain volume weight or test weight (TW), plant height (PH) and ear peduncle length (PdL). Based on the GY values reported in Maccaferri et al. (2011), each trial was classified according to its yield level as follows: low-yielding environment (LYE) ranging from 0.9 to 3.6 t ha^−1^, medium-yielding environment (MYE) ranging from 4.1 to 5.7 t ha^−1^ and high-yielding environment (HYE) ranging from 5.8 to 6.8 t ha^−1^. Each class included five environments, except LYEs for PH, PdL and TW where only three environments were considered. For each agronomic trait, single environment values and the general mean over all the tested environments (GMEs) were analyzed. The mean values of each environmental class were also included in the analysis (indicated as LYEs^M^, MYEs^M^ and HYEs^M^).

On average, in the field trials considered herein, the lines of this AM panel showed a heading window of 7 days, with the 70 % of the lines heading within 2 days and 80 % within 3 days (Maccaferri et al. 2011).

### Molecular profiling

In the present study, the SSR-based map (334 SSRs) reported in Maccaferri et al. (2011) was enriched with DArT marker. In total, 957 markers (334 SSRs and 623 DArT markers) were used for the molecular profiling of the 183 accessions.

DArT markers were generated by Triticarte Pty Ltd. (Canberra, Australia; http://www.triticarte.com.au). The durum wheat *Pst*I*/Taq*I array v 2.0, containing 7,600 single DArT clones obtained as described in Mantovani et al. (2008), was used for genotyping the panel. The locus designation used by Triticarte Pty. Ltd. was adopted (‘wPt’, ‘rPt’ and ‘tPt’ loci corresponding to wheat, rye and triticale clones, respectively), and alleles at polymorphic loci were scored as hybridization positive (1) or negative (0).

Markers were ordered according to a consensus map developed at the University of Bologna in the framework of an international cooperation for that purpose (Maccaferri et al. 2014a). Four mapping populations, i.e., Kofa × Svevo (KS RIL population, Maccaferri et al. 2008), Colosseo × Lloyd (CL RIL, Mantovani et al. 2008), Meridiano × Claudio (MC RIL, Maccaferri et al. 2011) and Simeto × Levante (SL RIL, Maccaferri et al. unpublished) were developed by the University of Bologna in collaboration with Produttori Sementi Bologna SpA (Argelato, BO, Italy). Ten additional maps provided by international partners were used to assemble a common consensus map, used to order the markers available for genotyping the experimental materials herein presented (Maccaferri et al. 2014a).

### Statistical analysis and association mapping analysis

The analysis of variance (ANOVA) was conducted on RSA traits based on the mean values of the experimental units. In order to reduce the effect due to blocks, the general mean of each set of genotypes included in the same block was used to correct the corresponding single values, using a linear regression method. To detect possible maternal effects due to seed size, an analysis of covariance was carried out for each trait using kernel weight as covariate.

Repeatability (*h* ^2^) was calculated on a mean basis across three replications. Accession means were used to calculate Pearson’s correlation coefficients of RSA traits versus the agronomic traits (GY, TKW, KPSM, PH and PdL) for each environment, as well as versus the mean values of each environmental class and the general mean.

To reduce the risk of false-positive marker-trait associations, rare alleles (i.e., with frequencies equal or <0.10) were considered as missing data. Additionally, marker points showing residual allelic heterogeneity within accession were also considered as missing data; thus, a total of 957 informative markers (i.e., 334 SSR and 623 DArT markers) that was possible to project on the consensus linkage map were utilized for Association Mapping (AM) analysis.

Presence of significant population structure in the panel had been previously shown by Maccaferri et al. (2011) with a combination of model- and distance-based analyses using the program STRUCTURE v. 2 (Pritchard et al. 2000). The optimal population structure model was identified by five hypothetical subgroups that led to the *Q* matrix of membership coefficients of each accession to all subgroups (for details see Maccaferri et al. 2011). Prior to proceeding with AM analysis, a multiple regression analysis was performed to test the significance of the differences among subgroups for the measured RSA traits.

A co-ancestry kinship (*K*) matrix was obtained for the mapped SSR and DArT markers by pair-wise genetic similarity values (GS_*ij*_) that were calculated for all accession pairs using the simple matching coefficient for multi-state markers. Linkage disequilibrium (LD) was estimated using the program TASSEL, v. 2.1 (www.maizegenetics.net, Yu et al. 2006); *D’* and *r* ^2^ values are a function of the corresponding inter-marker distances, and the comparison-wise significance was computed with 10,000 permutations. The *r* ^2^ LD value was estimated for intra-chromosomal loci and related to genetic distances between loci (cM). When all pairs of adjacent loci were in LD (*r* ^2^ > 0.3), this region was referred to as a LD block (Stich et al. 2005). Genome-wide scans for AM for both RSA traits and agronomic traits were conducted using the TASSEL program, ver. 4.0 (Bradbury et al. 2007). The 334 SSR and 623 DArT markers were tested for significance of marker-trait association under the fixed general linear model (GLM) including the *Q* population structure results as covariates (Q GLM), and the mixed linear model (MLM) including the *Q* population structure results plus the *K* kinship matrix (*Q* + *K* MLM).

For GLM analysis, besides the marker-wise association probability values, the experiment-wise association significance probability was obtained based on a permutation test implemented in TASSEL (10,000 permutations in total). The experiment-wise test provides a much more stringent threshold for significance as compared to the marker-wise test (Bradbury et al. 2007). Three significance levels of marker-trait association were considered, i.e., marker-wise at *P* = 0.01 [−log(P) = 2.0] and *P* = 0.001 [−log10(P) = 3.0] and experiment-wise at *P* = 0.1 [−log10(P) = 4.0, Bonferroni’s correction]. The QTL analysis was conducted on both RSA and agronomic traits.

In the present work, only RSA–QTLs co-locating with agronomic traits in at least two environments and/or on mean values (general means or at least one environmental class mean) are reported. Multiple, adjacent co-segregating significant markers were assigned to a unique QTL region if the strongest marker for the agronomic trait was within 2.5 cM from the reference marker (i.e., where the LOD value was highest) for RSA-QTLs, verifying a significant and strong LD among markers (possibly with *r* ^2^ values ≥0.6) (Massman et al. 2011). To facilitate the comparison of the effect of the same chromosomal region on different traits and assess their possible relationship, the effect of each single QTL was always referred to the reference allele (i.e., the allele with the highest frequency) as compared to the overall phenotypic mean at the RSA–QTL peak marker. The allele effect was also reported as percentage of the trait phenotypic mean.

## Results

### Phenotypic variation of the accessions’ panel for RSA traits

**Fig. 1:**
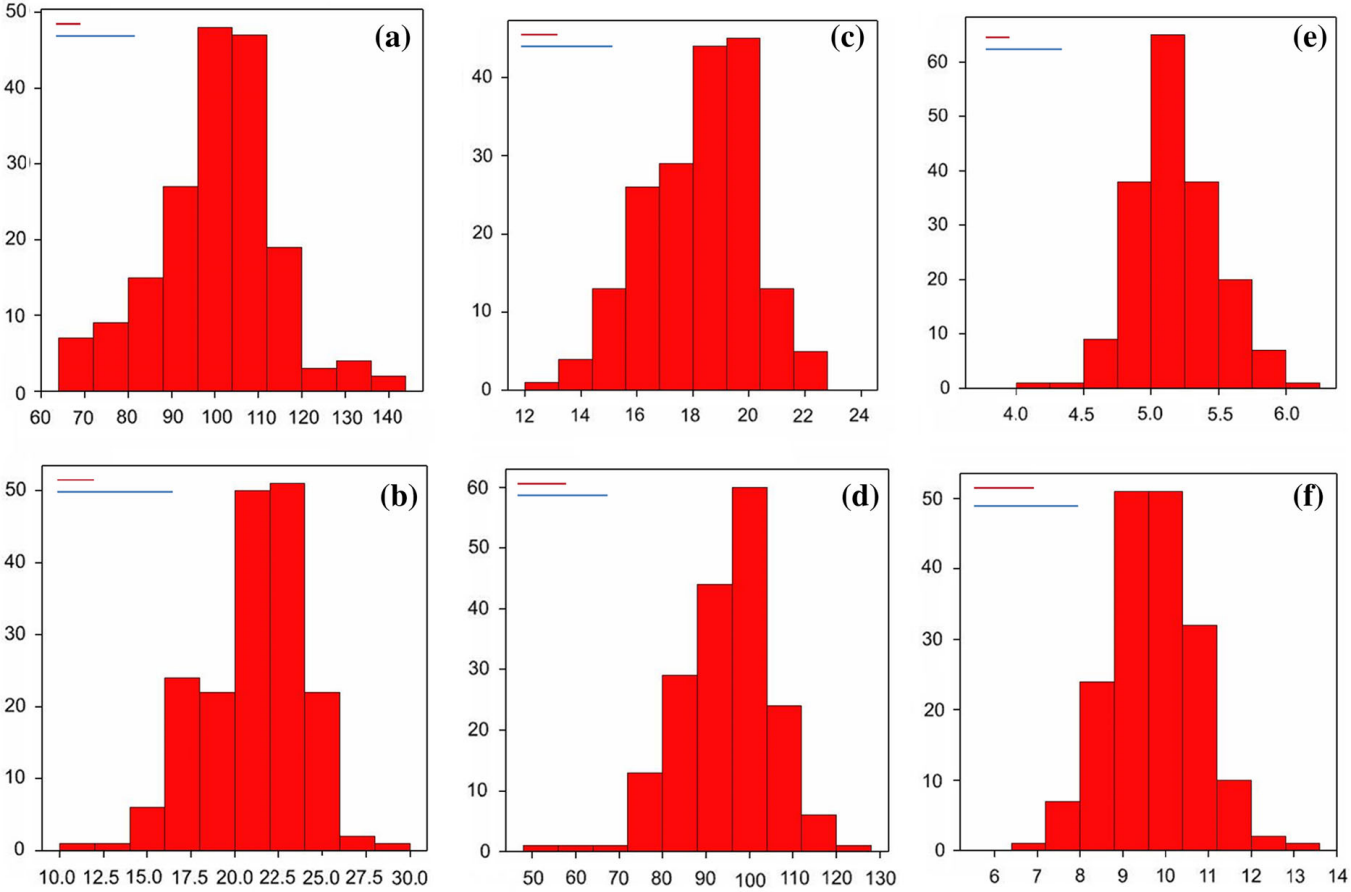
Frequency distribution of the RSA traits measured in the collection of 183 elite lines of durum wheat at the four-leaf growth stage. The *red line* at the *top* of each graph represents the standard deviation calculated on the check cultivar Meridiano. The *blue line* represents the LSD (*P* < 0.05) between accessions. **a** Seminal root angle (SRA, °). **b** Primary root length (PRL, cm). **c** Average root length (ARL, cm). **d** Total root length (TRL, cm). **e** Total root number (TRN, no.). **f** Shoot length (SL, cm). (Color figure online)

Frequency distributions for RSA traits are shown in Fig. 1, together with the standard deviation estimated on the check cultivar Meridiano, and the LSD based on the ANOVA results. All traits show an approximately normal distribution, indicating a polygenic control.

Kernel weight of the samples was taken into account as a covariate in the statistical analysis; the covariate was highly significant for SRA, ARL and TRL, while it was not significant for PRL, TRN and SL. The effect of the significant covariate was taken in due account in the calculation of the adjusted means of the corresponding traits. No significant regression (data not shown) between phenotypic values of RSA traits and population structure was detected, indicating that the variation observed herein was not influenced by the coefficient of membership of the tested material to the five germplasm subgroups.

The experimental material showed a wide range of variation for RSA traits as reported in Table 1. In detail, the RSA traits ranged as follows: SRA from 48° to 147° with a mean value of 100°, PRL from 13.8 to 32.9 cm with a mean value of 21.1 cm, TRL from 52.8 to 144.7 cm with a mean value of 94.6 cm, SL from 7.2 to 16.3 cm with a mean value of 9.7 cm and ARL from 12.0 to 26.0 cm with a mean value of 18.2 cm. TRN showed the lowest variation, from 4.01 to 6.46 roots per plant, with a mean value of 5.18. The ANOVA showed highly significant differences between the genotypes for all traits, with CV values ranging from 6.1 % for TRN to 17.0 % for PRL. Repeatability values ranged from 48.6 % for PRL to 72.8 % for SRA. In this respect, it should be underlined that these values are somehow overestimated, due to the fact that the genetic variance includes also the genotype by environment interaction.

**Table 1:** Mean, maximum and minimum values, ANOVA results and repeatability for the RSA traits and shoot length investigated at the four-leaf stage in seedlings of 183 durum wheat elite accessions

	SRA (°)	PRL (cm)	TRL (cm)	ARL (cm)	TRN (no.)	SL (cm)
Mean	100	21.1	94.6	18.2	5.18	9.70
Max	147	32.9	144.7	26.0	6.46	16.31
Min	48	13.8	52.8	12.0	4.01	7.20
Check (mean value)^a^	105	20.0	88.6	17.2	5.11	9.82
*P* accessions^b^	**	**	**	**	**	**
*P* replicates^c^	ns	ns	ns	ns	**	**
CV (%)	12.0	17.0	13.0	11.5	6.1	13.0
*h* ^2^ (%)	72.8	48.6	59.5	61.8	67.0	55.3
LSD (*P* < 0.05)	18.2	5.8	20.0	3.4	0.51	2.04

*SRA* seminal root angle, *PRL* primary root length, *TRL* total root length, *ARL* average root length, *TRN* total root number, *SL* shoot length, *CV* coefficient of variation, *h* ^2^ repeatability (mean basis),* LSD* least significant difference (*P* < 0.05)

^a^Meridiano, reference check line

^b^Significance of the difference between accessions

^c^Significance of the difference between replicates. ns = non significant

* *P* < 0.05; ** *P* < 0.01

### Correlation among RSA features and agronomic traits

The analysis of the correlations between RSA traits and agronomic performances of the 183 accessions is reported in Table 2. Correlation coefficients are reported for the mean values of each one of the three environmental classes, namely LYEs^M^, MYEs^M^ and HYEs^M^ as well as for the general mean over all environments (GMEs); additionally, the number of environments showing significant correlations within each class is reported in brackets. Highly significant albeit low correlations were detected between SRA and TKW (*r* = −0.23, −0.21 and −0.20 in MYEs^M^, HYEs^M^ and GMEs, respectively). Accordingly, highly significant and equally low correlations were detected between SRA and TW (*r* = −0.20, −0.26 and −0.22 in LYEs^M^, MYEs^M^ and GMEs, respectively). Moreover, SRA showed significant correlations with PdL in LYEs^M^ (*r* = −0.20), HYEs^M^ (*r* = −0.19) and GMEs (*r* = −0.20). All these correlations showed a significant, albeit low, negative value, thus suggesting that a more superficial root system (i.e., increase in SRA) is associated with a decreased PdL, TKW and TW. SRA was also significantly correlated with KPSM in MYEs^M^ (*r* = 0.23), HYEs^M^ (*r* = 0.24) and GMEs (*r* = 0.23). A significant, positive correlation was observed between SL and PH in MYEs^M^ (*r* = 0.19), HYEs^M^ (*r* = 0.21) and GMEs (*r* = 0.20).

**Table 2:** Correlation coefficient values and level of significance between root seminal traits (RSA) measured at the four-leaf stage with the agronomic traits measured in 15 field trials (see “Materials and Methods”), classified according to their average productivity levels, i.e., low, medium and high-yielding environments (LYE_s_^M^, MYE_s_^M^ and HYE_s_^M^, respectively), and with the general mean of environments (GME)

Agronomic traits	GY				TKW			
	LYE_s_^M^	MYE_s_^M^	HYE_s_^M^	GME_s_	LYE_s_^M^	MYE_s_^M^	HYE_s_^M^	GME_s_
*Correlation values among RSA traits and GY and TKW*								
RSA traits								
SRA		[1]^a^				−0.23** [2]	−0.21** [3]	−0.21*
PRL			[1]			[1]		
TRL	[1]		[1]					
ARL			[1]					
TRN	0.24** [1]			0.18*				
SL			[1]			[1]		

Agronomic traits	KPSM				TW			
	LYE_s_^M^	MYE_s_^M^	HYE_s_^M^	GME_s_	LYE_s_^M^	MYE_s_^M^	HYE_s_^M^	GME_s_
*Correlation values among RSA traits with KPSM and TW*								
RSA traits								
SRA		0.23** [3]	0.24** [3]	0.23**	−0.20* [1]	−0.26** [3]	[1]	−0.22**
PRL		[1]				[1]	[1]	
TRL	[1]							
ARL	[1]		[1]				[1]	
TRN	0.18 [1]					[1]		
SL		[1]						

Agronomic traits	PH				PdL			
	LYE_s_^M^	MYE_s_^M^	HYE_s_^M^	GME_s_	LYE_s_^M^	MYE_s_^M^	HYE_s_^M^	GME_s_
*Correlation values of RSA traits with PH and PdL*								
RSA traits								
SRA		[1]	[1]		−0.21* [1]	[1]	−0.19* [1]	−0.20*
PRL	[1]							
TRL								
ARL								
TRN								
SL		0.19* [1]	0.21** [3]	0.20*				

Traits are abbreviated as follows:* GY* grain yield,* TKW* thousand kernel weight,* KPSM* kernels per square mt,* TW* test weight,* PH* plant height,* PdL* peduncle length,* SRA* seminal root angle,* PRL* primary root length,* TRL* total root length,* ARL* average root length,* TRN* total root number,* SL* shoot length

^a^ The numbers reported in square brackets indicate in how many environments of each category a significant correlation was detected

As to GY, significant albeit low correlations were only detected with TRN in LYEs^M^ and GMEs (*r* = 0.24 and 0.18, respectively). No additional significant correlation was detected between GY and other RSA traits.

### QTL analysis for RSA features and agronomic traits

The results of AM analysis are reported in Table 3 and in Supplementary Table 1. QTLs are reported ordered according to their map position. In total, we identified 10 QTLs for SRA, 11 for PRL, 10 for ARL, 8 for TRL, 4 for TRN and 5 for SL. Among these 48 QTLs, 15 overlapped with QTLs for agronomic traits. Among these 15 QTLs, three (i.e., *QARL*_*1*_-*2A*, *QSRA*_*4*_-*6A* and *QSRA*_*6*_-*6B*) were significant at marker-wise significance level of *P* < 0.001 (−log10 > 3.0), while the other 12 were significant at the marker-wise significance level of *P* < 0.01 (−log10 > 2.0); none of these QTLs exceeded the experiment-wise threshold computed based on the Bonferroni’s correction, a highly conservative test as to Type I error. The QTLs described hereafter are identified according to the RSA traits for which the QTLs were detected; in case, the same QTL affected more than one RSA trait, the QTL is named after the trait showing the highest *P* value. The overlap with QTLs for GY, TW, TKW, KPSM, PH and PdL is also reported.

**Table 3:** QTLs with significant concurrent effects on RSA and agronomic traits and number of environments where the association was significant is reported. Peak positions related to a durum consensus map (see “Materials and Methods” for details)

QTL	Trait	Marker	Peak (cM)	*P* (−log10)	*R* ^2^ (%)	Effect (%)^a^	GY	TKW	KPSM	TW	PH	PdL
							(no. envs)	(no. envs)	(no. envs)	(no. envs)	(no. envs)	(no. envs)
*QSRA*_*1*_-*1B*	SRA	wPt-0655-1B	4.4	2.38	5.3	10.2	1 LYE			4 (1 LYE, 1 MYE, 2 HYE)		
*QPRL*_*1*_-*1B*	PRL	gwm124-1B-a2	109	2.32	6.1	7.9				**LYEs**, **MYEs**, **GMEs**		
*QARL*_*1*_-*2A*	PRL	cfa2263-2A-a2	73.7	2.13	5.5	−13.3			7 (3 LYEs, 2 MYEs, 2 HYEs), **GMEs**	2 (1 LYE, 1 MYE), **MYEs**, **GMEs**		**MYEs**
	ARL	cfa2263-2A-a2	73.7	3.25	9.4	−13.5						
	TRL	cfa2263-2A-a2	73.7	2.78	7.3	−14.1						
*QPRL*_*2*_-*2B*	PRL	barc183-2B-a2	67.1	2.88	5.8	−12.8	2 (1 LYE, 1 HYE)			2 HYE, **MYEs**, **GMEs**		
	ARL	barc183-2B-a2	67.1	2.56	5.2	−9.3						
	TRL	barc183-2B-a2	67.1	2.12	4.2	−9.3						
*QSL*_*1*_-*3A*	SL	wmc388-3A-a2	20.2	2.08	4.1	−5.3		1 HYE	1 HYE	1 HYE, **GMEs**		
*QTRN*_*1*_-*3A*	TRN	wmc428-3A-a6	48.4	2.23	5.1	−5.4		5 (1 MYE, 4 HYE), **HYEs**	8 (1 LYE, 3 MYE, 4 HYE), **MYEs**, **HYEs**, **GMEs**	2 (1 MYE, 1 HYE), **HYEs**	6 (2 MYE, 4 HYE)	2 (1 MYE, 1 HYE)
*QSRA*_*2*_-*3A*	SRA	barc1177-3A-a1	154	2.03	4.8	7.4				3 (2 MYE, 1 HYE)		
*QPRL*_*3*_-*4A*	PRL	wPt-2946-4A	88.3	2.19	4.4	10.3		1 HYE	2 (1 MYE, 1 HYE)			
*QSRA*_*3*_-*4B*	SRA	gwm888-4B-a2	29.6	2.18	4.7	−6.3	3 (2 LYE, 1 MYE), **LYEs**, **MYEs**, **GMEs**	1 MYE	1 MYE, **LYEs**		3 (1 MYE, 2 HYE), **HYEs**	1 LYE
*QTRN*_*2*_-*4B*	TRN	gwm6-4B-a6	85.4	2.04	5.5	3.5	1 HYE	3 (1 MYE, 2 HYE)	3 (2 MYE, 1 HYE)			
*QSRA*_*4*_-*6A*	SRA	gwm427-6A-a4	128	3.13	7.7	−10.0		5 (1 LYE, 2 MYE, 2 HYE), **LYEs**, **HYEs**		1 LYE, **LYEs**		
*QTRL*_*1*_-*6B*	TRL	wPt-7343-6B	14.3	2.13	5.3	6.2	**GMEs**	**LYEs**	1 MYE, **MYEs**		3 (1 MYE, 2 HYE), **MYEs**, **HYEs**, **GMEs**	4 (1 LYE, 1 MYE, 2 HYE), **HYEs**, **GMEs**
*QSRA*_*5*_-*6B*	SRA	wPt-6594-6B	21.5	2.31	4.5	6.2	2 HYE	**LYEs**		2 MYE		
*QSRA*_*6*_-*6B*	SRA	gwm1486-6B-a5	153	3.26	7.4	−8.8	2 (1 LYE, 1 HYE), **HYEs**		1 LYE			3 (1 LYE, 1 MYE,1 HYE), **GEMs**
*QARL*_*2*_-*7B*	ARL	gwm333-7B-a5	79.1	2.18	4.6	−10.8		4 (1 LYE, 2 MYE, 1 HYE)	1 HYE, **MYEs**, **GMEs**	2 (1 MYE, 1 LYE)		**LYEs**

Traits are abbreviated as follows:* GY* grain yield,* TKW* thousand kernel weight,* KPSM* kernels per square mt,* TW* test weight,* PH* plant height,* PdL* peduncle length, *SRA* seminal root angle,* PRL* primary root length,* TRL* total root length,* ARL* average root length,* TRN* total root number,* SL* shoot length

For each agronomic trait, the number (no. envs) and category of environments (i.e., low, medium or high-yielding environment: LYE, MYE and HYE, respectively) in which the QTL was detected is reported. Additionally, the acronyms **LYEs**, **MYEs**, **HYEs** and **GMEs** (in bold) indicate when an RSA QTL co-located with a QTL for agronomic traits based on the analysis of the mean values in low, medium and high-yielding environments and across all 15 environments, respectively. The allele effect has been computed as detailed in the last sentence of “Materials and Methods”

For the sake of clarity, we wish to point out that whenever the relative effects of RSA–QTL alleles on root traits were positively or negatively associated with the effects on grain yield and other agronomic traits, these concurrent effects are defined as ‘congruent’ and ‘contrasting’, respectively.

#### QTLs for seminal root angle

Among the 15 QTLs that overlapped with SRA–QTLs, six were identified for SRA on chromosomes 1B, 3A, 4B, 6A and 6B, with R^2^ values ranging from 4.59 (*QSRA*_*5*_-*6B*) to 7.74 % (*QSRA*_*4*_-*6A*). None of these QTLs for SRA co-located with QTLs for other RSA features measured in this study. Among these six SRA–QTLs, three (*QSRA*_*3*_-*4B*, *QSRA*_*5*_-*6B* and *QSRA*_*6*_-*6B*) co-located with GY–QTLs in at least two environments, while *QSRA*_*1*_-*1B* co-located with GY-QTLs in one environment only. *QSRA*_*3*_-*4B* co-located with GY-QTLs in three environments (two LYEs and one MYE), in LYE^M^, MYEs^M^ and GMEs. Notably, the effects estimated for GY were congruent with those estimated for SRA. *QSRA*_*5*_-*6B* co-located with GY in two HYEs; the GY effects were congruent with those estimated for SRA. *QSRA*_*6*_-*6B* co-located with GY in two environments (one LYE and one HYE) and in HYEs^M^; in this case, the allelic effects for SRA and GY were congruent in one HYE and HYEs^M^ but contrasting in the LYE.

Considering GY components (i.e., TKW and KPSM), four out of the six SRA–QTLs (all except *QSRA*_*1*_-*1B* and *QSRA*_*2*_-*3A*) co-located with QTLs for at least one of these traits in two or more environments. In detail, *QSRA*_*3*_-*4B* co-located with KPSM in one MYE (with contrasting effects in comparison with SRA) and in LYEs^M^, with an affect congruent with SRA; moreover, the same QTL co-located also with TKW in one HYE, with an effect in contrast to SRA. *QSRA*_*4*_-*6A* co-located with TKW in five environments (one LYE, two MYEs and two HYEs) as well as with TKW in LYEs^M^ and HYEs, all showing effects congruent with those on TKW and SRA. *QSRA*_*5*_-*6B* co-located with TKW in LYEs^M^ with a consistent effect for TKW and SRA; even though this QTL overlapped with LYEs^M^ only, it is noteworthy because its effects on the mean of five environments suggests a more prominent role for this QTL. *QSRA*_*6*_-*6B* overlapped with QTLs for KPSM in one LYE and in one MYE, both of which showed contrasting effects as compared to SRA.

Considering the other agronomic traits, two RSA–QTLs appear particularly interesting for their co-location with QTLs for TW, a trait closely related to grain quality and to starch accumulation capacity in the final phase of grain filling in durum wheat. *QSRA*_*1*_-*1B* co-located with TW-QTLs in four environments (one LYE, one MYE and two HYEs), all with contrasting effects as compared to SRA except for one HYE (Itl1-r04), where consistent effects were noted, Additionally, this QTL influenced GY in one LYE, with contrasting effects as compared to SRA. Moreover, *QSRA*_*2*_-*3A* influenced TW in three environments, two MYEs (with effects congruent with SRA) and one HYE (with an effect contrasting with that on SRA). *QSRA*_*4*_-*6A* influenced TW in one LYE and in LYEs^M^, with contrasting effects with those on SRA in both cases. In two MYEs, TW was influenced also by *QSRA*_*5*_-*6B*, in both cases with congruent effects on TW.

Finally, two SRA–QTLs co-located with QTLs for morphological traits at the adult stage of the plants in the field. *QSRA*_*3*_-*4B* influenced PH in three environments (two HYEs and one MYE) and in HYEs^M^, in all cases with contrasting effects on PH. Moreover, *QSRA*_*3*_-*4B* influenced PdL in the least productive LYE (Spn2-r05), also in this case with an effect in contrast to those on SRA. *QSRA*_*6*_-*6B* showed contrasting effects on PdL in three environments (one LYE, one MYE and one HYE).

#### QTLs for total root number

Two QTLs for TRN (*QTRN*_*1*_-*3A* and *QTRN*_*2*_-*4B*) overlapped with QTLs for agronomic traits. *QTRN*_*1*_-*3A* (*R* ^2^ = 5.10 %) co-located with KPSM–QTLs in eight environments (four HYEs, three MYEs and one LYE) as well as in HYEs^M^, MYEs^M^ and in GMEs, in all cases with effects congruent with those on TRN. Moreover, *QTRN*_*1*_-*3A* co-located with TKW–QTLs in five environments (four HYEs and one MYE) and in HYEs^M^, in all cases with contrasting effects as compared to those on TRN. *QTRN*_*1*_-*3A* co-located also with TW–QTLs in two environments (one HYE and one MYE with contrasting and congruent effects, respectively) and in HYEs^M^ (with contrasting effects). Finally, it overlapped with PH–QTLs in six environments (four HYEs and two MYEs) and with PdL–QTLs in two environments (one MYE and one HYE) in all cases with contrasting effects.

*QTRN*_*2*_-*4B* (*R* ^2^ = 5.59 %) co-located with TKW–QTLs in two HYEs (with contrasting effects) and one MYE (with congruent effects). *QTRN*_*2*_-*4B* co-located also with KPSM–QTLs in three environments (with contrasting effect in two MYEs and congruent effects in one HYE). *QTRN*_*2*_-*4B* also co-located with a GY–QTL in one HYE environment, with contrasting effects.

#### QTLs for primary, total and average root length

Among the six QTLs that were identified for ARL, PRL, and TRL, two (*QARL*_*1*_-*2A* and *QPRL*_*2*_-*2B*) influenced all three traits (the identification acronym identifies the trait with the highest *R* ^2^ value), while the other four QTLs were specific for only one of these RSA traits. *QARL*_*1*_-*2A* was identified on chromosome 2A, with *R* ^2^ values of 9.41 % for ARL, 7.33 % for TRL and 5.56 % for PRL. In all cases, the reference allele showed negative effects for these RSA traits. *QARL*_*1*_-*2A* influenced KPSM in seven environments (three LYEs, two MYEs and two HYEs) and in GMEs, in all cases with congruent effects. Moreover, *QARL*_*1*_-*2A* co-located with TW–QTLs in two environments (one LYE and one MYE), in MYEs^M^ and in GMEs, in both cases with contrasting effects. *QPRL*_*2*_-*2B* was detected on chromosome 2B, with *R* ^2^ values equal to 5.83 % for PRL, 5.25 % for ARL and 4.21 % for TRL. This QTL showed a congruent effect on GY in one LYE and a small contrasting effect in one HYE. Additionally, it co-located with TW–QTLs in two HYEs, in MYEs^M^ and in GMEs, always with congruent effects.

Considering the scored RSA traits, PRL was influenced by *QPRL*_*1*_-*1B* and *QPRL*_*3*_-*4A* with *R* ^2^ values of 6.18 and 4.47 %, respectively, both showing congruent effects. *QPRL*_*1*_-*1B* co-located with TW–QTLs in LYEs^M^ (with a congruent effect) as well as in HYEs^M^ and in GMEs (in both cases with contrasting effects). *QPRL*_*3*_-*4A* co-located with KPSM–QTLs in two environments (one HYE and one MYE, both with congruent effects) and with TKW–QTLs in the same HYE (Itl2-r04) with contrasting effects.

Considering TRL, *QTRL*_*1*_-*6B* (*R* ^2^ = 5.32 %) co-located with a GY–QTL in GMEs (with a congruent effect) and with a TKW–QTL in LYE^M^ (with a contrasting effect). Moreover, it co-located with KPSM–QTLs in one MYE and in MYEs^M^, in both cases with consistent effects. Additionally, it co-located with PH–QTLs in three environments as well as in MYEs^M^, HYEs^M^ and GMEs, and with PdL–QTLs in four environments, HYEs^M^ and GMEs. At this QTL, the reference allele negatively affected both PH and PdL while affecting positively TRL.

As to ARL, *QARL*_*2*_-*7B* (*R* ^2^ = 4.67 %) co-located with TKW in four environments (one LYE, two MYEs and one HYE), with contrasting effects; moreover, *QARL*_*2*_-*7B* co-located with TW in two environments (one LYE with a consistent effect and one MYE with a contrasting effect). Additionally, it co-located with KPSM–QTLs in one HYE, in MYEs^M^ and in GMEs, in all cases with effects congruent with those on ARL. Finally, it co-located with one PdL–QTL in LYEs^M^, showing a contrasting effect.

#### QTLs for shoot length

Only one QTL identified for SL co-located with agronomic traits. *QSL1*-*3A* (*R* ^2^ = 4.14 %) co-located with TW–QTLs in one HYE and in GMEs showing consistent positive effects in both cases but contrasting with those on SL. Additionally, *QSL1*-*3A* co-located with QTLs for KPSM and TKW in one HYE, with a congruent effect on SL, and a congruent one on TKW.

## Discussion

A valuable feature of the panel of genotypes evaluated in this study is their limited range in heading time as previously reported (Maccaferri et al. 2011). Limited variability in phenology is of utmost importance for a meaningful interpretation of studies to investigate the role of drought-adaptive features on field performance across environments characterized by large variability in soil moisture during the reproductive stage, a factor that plays a key role in setting yield potential particularly in Mediterranean environments (Araus et al. 2003a, b; Garcia del Moral et al. 2003; Royo et al. 2010).

### Phenotypic variation for RSA traits

A number of approaches/techniques have been developed for the description of RSA in controlled environments at different levels of throughput and cost (Tuberosa et al. 2002; Sanguineti et al. 2007; Nagel et al. 2009; Zhu et al. 2011; Grossman and Rice 2012; Pacheco-Villalobos and Hardtke 2012; Postma and Lynch 2012; Bai et al. 2013; Lavenus et al. 2013; Watt et al. 2013; Wasson et al. 2014). The approach utilized herein allows for a reasonably rapid and accurate phenotyping of RSA in hundreds of plants, as usually required by any QTL study.

With the exception of TRN, the durum accessions tested herein have shown a range of variation (from two up to three fold in magnitude) and repeatability (from 48.6 % for PRL to 72.8 % for SRA) for RSA traits that appears suitable for further investigation. These results are particularly noteworthy considering that the tested materials are mainly elite cultivars that usually explore only a limited portion of the variability present in the genepool available for each species. The variability found for RSA features may to a certain extent reflect the adaptive value of such features for the environmental conditions prevailing in the original selection sites of each cultivar. Therefore, this experimental material provides further opportunities for dissecting RSA complexity and its possible functional role in field performance and grain yield plasticity of durum wheat.

### Correlation among RSA features and agronomic traits

Overall, the correlations between RSA features and agronomic traits were very low, not at all unexpectedly in consideration that RSA data were measured at a very early stage and in growing conditions unable to properly mimic soil conditions, hence unable to account for RSA plasticity and its adaptive role for grain yield (GY) in the field. This notwithstanding, once the variability of phenotypic values was dissected at the QTL level, the analysis of RSA data and agronomic performance has revealed several concurrent QTL effects on RSA, GY and other agronomic traits. Other studies conducted in maize (Landi et al. 2007, 2010) and rice (Steele et al. 2007; Uga et al. 2013) grown under controlled conditions have revealed sizeable, concurrent effects of QTLs for RSA features on GY and other agronomic traits evaluated under field conditions, thus providing valuable opportunities for genomics-assisted breeding approaches, like in the case of rice (Steele et al. 2006).

Among the investigated root traits, SRA was negatively correlated with TKW and TW, a result possibly due to the influence of root angle on root distribution in soil layers, hence on water uptake from deeper soil horizons (Manschadi et al. 2010; Lynch 2013; Lynch et al. 2014). SRA was also correlated with both KPSM (positive association) and TKW (negative association) in MYEs^M^, HYEs^M^ and GMEs. These findings account for the lack of association of SRA with GY since a counterbalancing effect between the two main yield components inevitably leads to a lack of significant effects of such variability on GY itself.

The positive, albeit low, correlation observed between TRN and GY in LYEs^M^ and also GMEs suggests a beneficial adaptive role of TRN on GY in environments with low yield potential due to unfavorable growth conditions, consistently with the study conducted by Liu et al. (2013) on RSA traits and GY in wheat at two different water regimes. Notably, among the RSA features herein investigated TRN was the trait with the highest correlation with GY. These results could be ascribed to the fact that a higher number of seminal roots provide greater early vigor a trait known to be particularly crucial for enhancing water uptake in drought-prone environments (Blum 1996; Richards 2006, 2008; Reynolds and Tuberosa 2008). It is noteworthy that in the study conducted by Liu et al. (2013), focusing on RSA traits and GY at two different water regimes, TRN was the trait with the highest correlation with GY. Accordingly, we observed a positive correlation between SL and PH in MYEs^M^, HYEs^M^ and GMEs, a result that further underlines the importance of early seedling growth on yield performance of wheat.

### QTL analysis for RSA features and agronomic traits

The large number of QTLs (48 in total) for RSA features evidenced in our study underlines the complexity of the genetic control of these traits already at an early growth stage. Previous QTL studies conducted on the same set of genotypes considered herein have revealed striking differences as to the role of specific QTLs on specific traits when the genetic dissection was based upon biparental mapping (Maccaferri et al. 2008; Graziani et al. 2014) and association mapping (Maccaferri et al. 2011). Therefore, a more exhaustive search for novel haplotypes governing RSA traits in durum wheat should deploy larger and more genetically diverse panels as well as biparental mapping populations, preferably derived from non-elite materials such as landraces and wild relatives (e.g., emmer wheat and *T. dicoccoides*) more likely to carry novel alleles for RSA features conferring adaptation to water-limited conditions. The use of high-density SNP maps (Trebbi et al. 2011; Van Poecke et al. 2013; Maccaferri et al. 2014a, b) coupled with sequencing information will facilitate the identification of novel haplotypes and in some case may also provide valuable clues on the possible candidates underlying root phenotypes. Along this line, the high LD of elite durum wheat germplasm (Maccaferri et al. 2005, 2006) does not allow for meaningful speculation on the possible role of genes syntenic to candidates that have been suggested to control RSA features in other cereals.

Approximately, 30 % (15/48) of the SRA–QTLs concurrently affected agronomic traits including also GY and/or its main components, thus providing circumstantial albeit valuable evidence as to the implications of RSA variability at an early growth stage on the field performance of durum wheat.

The RSA trait with the most extensive overlap with agronomic performance was SRA, a feature of particular interest in both durum and bread wheat as recently highlighted by Christopher et al. (2013) since the angle of roots at their emergence from the seeds could be a valuable proxy for rooting depth (Kato et al. 2006; Wasson et al. 2012). Accordingly, modeling of RSA features suggests that a narrow angle of wheat roots could lead, in general, to deeper root growth and higher yields (de Dorlodot et al. 2007; Manschadi et al. 2008; Wasson et al. 2012; Lynch 2013). In the present study, considering the results obtained for the single QTLs, the relationship between GY, GY component traits and SRA varied according to the level of yield potential of each particular location, consistently with the findings of Christopher et al. (2013) in bread wheat, thus indicating that the optimal root angle ideotype is likely to vary according to the target environment. Other studies have underlined the specificity of the response of GY to RSA features in different environments. As reported by Wasson et al. (2012), in wheat, the same RSA features led to markedly different GY responses according to the environment in which those materials were first selected and then cultivated (Oyanagi et al. 1993; Manschadi et al. 2008). Therefore, if experimental evidence suggests that SRA in seedlings might be closely related to adult plant rooting depth, the field conditions in which the crop is grown determine the final performance in a given environment (White and Kirkegaard 2010; Wasson et al. 2012). In the present study, the six QTL regions that influenced SRA and agronomic performance showed contrasting relationships as to the effects of SRA on GY and its components. Contrasting effects of a specific drought-adaptive QTL on GY as a function of different environmental conditions have been previously reported, and the underlying reasons critically discussed (Collins et al. 2008). In this respect, particularly noteworthy is the case of *QSRA*_*6*_-*6B*, where SRA and GY effects were negatively associated in Spn2-r05, a LYE devoid of moisture in the superficial soil horizon (Maccaferri et al. unpublished) usually more massively explored by root systems with a wider SRA. Conversely, SRA and GY effects at *QSRA*_*6*_-*6B* were positively associated in HYEs^M^, possibly due to the fact that shallow roots have been shown to more effectively acquire mobile and immobile nutrients that in fertile soils tend to be more abundant in topsoil layers (Lynch 2013). Notably, a PH–QTL has been mapped to the same position in durum wheat (Sanguineti et al. 2007), a finding consistent with the effects of the same region reported in the present work for PdL, the main component of PH in durum wheat (Maccaferri et al. 2008). A similar relationship between SRA with GY and TW was observed for *QSRA*_*1*_-*1B*, where SRA was negatively related to GY in a LYE and to TW in one environment of each one of the three yield classes (LYE, MYE, HYE); however, a positive association with the QTL effects on TW was observed in P3r04, the second highest yielding environment. At the other four SRA–QTLs, the effects on SRA and GY–QTL were positively related. Among these four QTLs, *QSRA*_*3*_-*4B* showed a negative association of SRA with PH mainly in HYEs as well as with PdL in Spn2-r05, a LYE. Interestingly, *QSRA*_*3*_-*4B* co-located with a QTL identified by Ren et al. (2012) for root length-related traits in bread wheat, thus highlighting the importance of this region in governing RSA in both species and making this QTL a valuable candidate for fine mapping and cloning.

In our study, also *QSRA*_*4*_-*6A* showed concurrent effects on TKW and SRA in P4r05 (i.e., the environment with the lowest yield) and LYEs^M^, again suggesting a positive role of a potentially deeper root systems in drier environments. This hypothesis is further supported by the co-location of *QSRA*_*4*_-*6A* with the QTL identified in durum wheat by Kubo et al. (2007) for penetration ability of the root in deeper soil layers, consistently with the root ideotype proposed by Lynch (2013) as a means to allow the plant to more effectively explore deeper soil levels and capture larger amounts of soil moisture.

Among the QTLs detected for RSA traits and overlapping with agronomic features, six were related to root length. In general, at these QTLs, a positive association between root length and agronomic performance was observed, mainly in environments with lower water availability.

## Conclusions

Notwithstanding the critical role played by roots on the agronomic performance of wheat, so far only two studies have addressed the implications of RSA–QTLs of seedlings to field performance in wheat (Sanguineti et al. 2007; Bai et al. 2013). Our study has unveiled the presence of several novel RSA–QTLs while highlighting those with concurrent effects also on agronomic traits and yield under field conditions. Among RSA traits, seminal root angle appears the most promising for undertaking further studies on the role of RSA on field performance. Based upon the results herein reported, we have developed biparental RIL populations obtained from the cross of accessions contrasted for root angle and other RSA features in order to more accurately assess the genetic basis of RSA in durum wheat and the effects of the most relevant RSA–QTL haplotypes on GY in different water regimes. Eventually, this information might lead to the identification of RSA loci worthy of a MAS approach aimed to enhance yield potential and yield stability of durum wheat grown under different soil moisture conditions.

## Electronic supplementary material

Below is the link to the electronic supplementary material.

Supplementary material 1 (XLSX 27 kb)

## Abbreviations

AM: Association mapping
ARL: Average root length
DArT^®^: Diversity Array Technology^(R)^ markers
GME_s_: General mean of environments
GY: Grain yield
HYE: High-yielding environments
HYE_s_^M^: Mean of high-yielding environments
KPSM: Number of kernels per square meter
LD: Linkage disequilibrium
LYE: Low-yielding environment
LYE_s_^M^: Mean of low-yielding environments
MAS: Marker-assisted selection
MYE: Medium-yielding environment
MYE_s_^M^: Mean of medium-yielding environments
PdL: Ear peduncle length
PH: Plant height
PRL: Primary root length
QTL: Quantitative trait locus
RIL: Recombinant inbred line
RSA: Root system architecture
SL: Shoot length
SRA: Seminal root angle
SSR: Simple sequence repeat markers
TKW: Thousand kernel weight
TRL: Total root length
TRN: Total number of roots
TW: Test weight (grain volume weight)
